# An adaptive random search for short term generation scheduling with network constraints

**DOI:** 10.1371/journal.pone.0172459

**Published:** 2017-02-24

**Authors:** J. A. Marmolejo, Jonás Velasco, Héctor J. Selley

**Affiliations:** 1 Faculty of Engineering, Anahuac University, Mexico-City, Mexico; 2 CONACYT–Center for Research in Mathematics, Aguascalientes, Mexico; IUMPA - Universitat Politecnica de Valencia, SPAIN

## Abstract

This paper presents an adaptive random search approach to address a short term generation scheduling with network constraints, which determines the startup and shutdown schedules of thermal units over a given planning horizon. In this model, we consider the transmission network through capacity limits and line losses. The mathematical model is stated in the form of a Mixed Integer Non Linear Problem with binary variables. The proposed heuristic is a population-based method that generates a set of new potential solutions via a random search strategy. The random search is based on the Markov Chain Monte Carlo method. The main key of the proposed method is that the noise level of the random search is adaptively controlled in order to exploring and exploiting the entire search space. In order to improve the solutions, we consider coupling a local search into random search process. Several test systems are presented to evaluate the performance of the proposed heuristic. We use a commercial optimizer to compare the quality of the solutions provided by the proposed method. The solution of the proposed algorithm showed a significant reduction in computational effort with respect to the full-scale outer approximation commercial solver. Numerical results show the potential and robustness of our approach.

## Introduction

A definition of Short Term Generation Scheduling (Unit commitment) used in Smart grid operations is a scheduling problem with two objectives: The power dispatch that requires distributing the system load to the generating units over a given time horizon and the start-up and shutdown schedules of the power output generators.

The planning horizon in the short term usually lasts from 24 to 168 hours (see for instance [1]). Additionally, the Short Term Generation Scheduling solves a very large-scale, time-varying, non-convex, mixed-integer optimization problem. For these reasons, this problem belongs to the set of NP-hard problems.

The Short Term Generation Scheduling (STGS) with network constraints in an NP-hard Mixed Integer Non Linear Problem. We readily know that exacts methods are inefficient for large power systems (see [2–5]). Because of this, we present an alternative for generating good solutions in short computing time based on an Adaptive Random Search strategy.

One advantage when the Lagrangian relaxation is used is to eliminate constraints that complicate the structure of the original problem. Because in our work we use Lagrangian relaxation, the model becomes drastically simplified and this allows to use the AGS algorithm in a natural way.

Lagrangian relaxation (LR) is the most widely used method to solve STGS by relaxing some constraints [6, 7]. This method is based on the duality theory, and tries to find optimal dual variables that maximize the dual Lagrangian.

A way to effectively implement LR is to apply “divide and conquer principle”, that is, to decompose the STGS into a master problem and several manageable subproblems to be solved separately. The sub-problems are linked by Lagrange multipliers that are added to the master problem to yield a dual problem. The dimensionality of such dual problem makes it easier to solve than the primal. The multipliers are updated through different techniques. A popular choice is the use of any subgradient method, but the mayor difficulty associated with this option is the feasibility of the initial solution. This phenomenon is due to the dual nature of the algorithm.

The commitment states obtained in LR generally are infeasible because STGS is a MINLP (Mixed Integer Non-Linear Programming) problem. The duality gap is an inherent disadvantage of this technique, i.e. the dual solution may be far away from the optimal solution (see [8]). The feasible commitment states are obtained after adjustment. Usually heuristic methods are needed to modify the dual solution obtained for LR into a feasible solution. The ways to obtain these feasible solutions may vary notably.

Moreover, the most used random search algorithms to solve STGS are Evolutionary Programming (EP) [9, 10], Genetic Algorithm (GA) [11, 12], Simulated Annealing (SA), [13, 14] Particle Swarm Optimization (PSO) [15] and Tabu Search (TS) [16]. Genetic algorithm and particle swarm optimization have become increasingly popular in recent years in science and engineering disciplines. They are attracting much attention, because of their great potential for modeling engineering problems [17–19].

For instance, in [17] GA is used to identify the model parameters for an Ultracapacitor, based on time-domain data. In [18] GA is employed to extract the optimal model parameters based on the Hybrid Pulse Power Characterization (HPPC). Finally in [19] a GA is proposed for effectively achieving optimal component sizing of a hybrid energy storage system in an electric vehicle.

In [15] a new approach via multi-particle swarm optimization (MPSO) to solve the unit commitment (UC) problem is presented meanwhile in [13, 14] a simulated annealing algorithm (SAA) is presented to solve UC.

However the obtained results by EP, GA, SA, PSO and TS required a considerable amount of computational time especially for a large system size. This kind of techniques are not suitable for STGS problem due its NP-hard nature.

Because the main objective of an effective method for solving UC with LR is how to obtain feasible solutions. We present an alternative for generating feasible solutions in short computing time. Our proposal is based on an Adaptive Gibbs Sampling (AGS) algorithm.

The random search is one of the pillars of most heuristic methods to solve engineering optimization problems. The success of these methods to find good solutions (near-optimal) in an optimization problem is mainly achieved by tuning parameters. The perturbation of a parameter makes it possible to explore large regions of a landscape and potentially escape from local optima, resulting in the exploration of different local optima. The GA introduces a stochastic perturbation in their mutations [20], while SA presents these perturbations through their temperature levels and the cooling task [21]. For instance, a bad selection of the perturbation parameters in these methods could be result in a large risk of getting trapped in local regions. This fact points out of a need for an accurate selection of the step size parameters which dictate the amount of noise in the random search. On the other hand, the optimal scale of this perturbation, in order to achieve a good balance between exploration and exploitation, depends on the shape of the search landscape associated with the optimization problem. Therefore, this dependence makes the parameter selection a major issue in the design of heuristic algorithms.

In this study, we present an adaptive random search approach based on the Markov Chain Monte Carlo method to address a short term generation scheduling with network constraints. The key to the proposed method is that the noise level of the random search can be adaptively controlled according to the landscape. Thus, the noise intensity allows exploring the entire search space, and noise-reduction allows exploitation in the promising regions where local optima exist. The algorithm is well described and compared in unconstrained global optimization problems by [22]. The effectiveness and robustness of this method was proved in several complex problems in order to find reasonable quality solutions for global optimization problems. Motivated by the success of the performance of the method over complex problems, we decided to apply our method in this engineering optimization problem. Besides, we use a full-scale outer approximation commercial solver to compare the quality of the solutions provided by the proposed method.

This paper is organized as follows: in the following section we present the problem formulation of STGS. In section “Solution Methodology”, we introduce a novel optimization method which we call AGS algorithm based on infeasible solutions calculated for LR algorithm. We present our computational experience in the section this called, additionally we show our results on three test systems. Finally we draw our conclusions and summarize future work in the last section.

## Problem formulation

The STGS with network constraints problem consists in determining the mix of generators and their estimated output level to meet the expected demand of electricity over a given time horizon (a day or a week), while satisfying the load demand, spinning reserve requirement and transmission network constraints.

In this work, we address a STGS based on the notation presented in [23], where network constraints are represented through a DC model (see [1]) and we consider a multi-period time horizon. The objective is to minimize a function that includes fixed costs, start-up costs and operating costs. A second order polynomial describes the variable costs as a function of the electric power. The following notation is used in the mathematical model:

**Sets:**

*J* Set of indices of all power plants.
*K* Set of period indices.
*N* Set of indices of all nodes.
Λ_*n*_ Set of indices of the power plants *j* at node *n*.
Ω_*n*_ Set of indices of nodes connected and adjacent to node *n*.

**Constants:**

*A*_*j*_ Start up cost of power plant *j*.
*B*_*nm*_ Subsceptance of line *n* − *m*.
*C*_*nm*_ Transmission capacity limit of line *n* − *m*.
*D*_*nk*_ Load demand at node n during period *k*.
*E*_*j*_(*t*_*jk*_) Nonlinear function representing the operational cost of power plant *j* as a function of its power output in period *k*.
*F*_*j*_ Fixed cost of power plant *j*.
*K*_*nm*_ Conductance of line *n* − *m*
*R*_*k*_ Spinning reserve requirement during period *k*.
$\bar{T_{j}}$ Maximum power output of plant *j*.
$\underline{T_{j}}$ Minimum power output of plant *j*.
*nr* Reference node with angle zero.

**Decision variables:**

*t*_*jk*_ Power output of plant *j* in period *k*.
*δ*_*nk*_ Angle of node *n* in period *k*.
$v_{jk}=\{\begin{array}{cc} 1 & \;\text{if}\;\text{plant}\;j\;\text{is}\;\text{committed}\;\text{in}\;\text{period}\;k,\\ 0 & \;\text{otherwise}\; \end{array}$
$y_{jk}=\{\begin{array}{cc} 1 & \;\text{If}\;\text{plant}\;j\;\text{is}\;\text{started}\;\text{up}\;\text{at}\;\text{the}\;\text{beginning}\;\text{of}\;\text{period}\;k,\\ 0 & \;\text{otherwise}\; \end{array}$

**Objetive function:**

  $$\begin{array}{c} \text{min}\;Z=\sum_{k\in K}\sum_{j\in J}[F_{j}v_{jk}+A_{j}y_{jk}+E_{j}\left(t_{jk}\right)]. \end{array}$$ (1)

**Constraints:**

**Load balance**
  $$\begin{array}{c} \sum_{j\in \Lambda_{n}}t_{jk}+\sum_{m\in \Omega_{n}}B_{nm}[\delta_{mk}-\delta_{nk}]-\sum_{m\in \Omega_{n}}K_{nm}[1-\cos(\delta_{mk}-\delta_{nk})]=D_{nk},\;\\ \forall n\in N,\forall k\in K. \end{array}$$ (2)
**Spinning reserve**
  $$\begin{array}{c} \sum_{j\in J}\bar{T_{j}}v_{jk}\geq \sum_{n\in N}D_{nk}+R_{k},\;\forall k\in K. \end{array}$$ (3)
**Generation limit**
  $$\begin{array}{c} \underline{T_{j}}v_{jk}\leq t_{jk}\leq \bar{T_{j}}v_{jk},\;\forall j\in J,\forall k\in K. \end{array}$$ (4)
**Transmission capacity limits**
  $$\begin{array}{c} -C_{nm}\leq B_{nm}[\delta_{mk}-\delta_{nk}]\leq C_{nm},\;\forall n\in N,\forall k\in K,\forall m\in \Omega_{n}. \end{array}$$ (5)
**Start-up and shut-down of power units**
  $$\begin{array}{c} y_{jk}\geq v_{jk}-v_{jk-1},\;\forall j\in J,\forall k\in K\backslash \left\{1\right\}. \end{array}$$ (6)
**Angular limit voltage**
  $$\begin{array}{c} -\pi \leq \delta_{nk}\leq \pi ,\;\forall n\in N\backslash \left\{nr\right\},\forall k\in K. \end{array}$$ (7)

The objective Eq (1) is to minimize the start up cost *A*_*j*_*y*_*jk*_ and the operating cost of each plant. The operating cost of each plant *j* includes a fixed cost *F*_*j*_*v*_*jk*_ and a variable cost *E*_*j*_(*t*_*jk*_). There is a power balance Constraint (2) per node and time period. In each period, the production has to satisfy the demand and losses in each node. Power line losses are modeled through cosine approximation and it is assumed that the demand for electric energy is known, and is discretized into *t* periods. There are many approximations to model power line losses, some of them are linear, and some are non-linear. Further details of the cosine approximation can be found in [24]. Spinning reserve requirements are modeled in Eq (3). In each period the running units have to be able to satisfy the demand and the pre-specified spinning reserve. In Eq (4), each unit has a technical lower and upper bounds for the power production. The transmission capacity limits of lines in Eq (5) serve the purpose of avoiding problems in the dynamic stability of the system. The Constraint (6) describes how the units start-up, run and shut-down (a running unit cannot be started-up). Finally, the angle in all buses has lower and upper bounds given by Eq (7).

## Solution methodology

In this paper, we extend the AGS algorithm (see [22]) for continuous optimization to tackle mixed-variable optimization problems. First, the LR was used just for bounding purposes, and then the AGS algorithm was used to construct feasible solutions in reasonable computational times.

### Lagrangian relaxation framework

Because STGS is an NP-Hard problem, the global optimal solution cannot be obtained for large scale power systems. For this reason, we use LR to calculate a lower bound of the optimal solution. The main disadvantage of this method is the difference between primal and the dual solutions, which is the duality gap. This situation determines that solutions obtained by LR are infeasible for the original problem.

LR is based on the duality theory, which tries to find optimal dual variables that maximize the dual Lagrangian. These dual variables (Lagrangian multipliers) need to be updated in order to improve the lower bound. In this work, we use subgradient method to update the lagrangian multipliers. The parameters of the subgradient and other especifications are shown in [25].

For LR we decomposed the STGS problem into *n* subproblems, one per generation node [25]. DICOPT solver (see [26]) was used to maximize the Lagrangian bound. The LR algorithm was used just for bounding purposes. Since the main objective of an effective method for solving STGS with LR is to obtain feasible solutions, we present an alternative to constructing feasible solutions in short computing time based on the AGS algorithm.

In this paper, the objective function used by the AGS algorithm does not consider an explicit mechanism for handling constraints. For this reason we use the LR in the original problem. All of the system constraints are dualized in original objective function. This manipulation is necessary due to fact that AGS needs a function without constraints.

Applying Lagrange duality to the Constraints (2), (3) and (5) in STGS yields:

**Dual Function:**

$$\begin{array}{c} \max_{\lambda_{k}\geq 0,\mu_{k}\geq 0,\gamma_{k}\geq 0,\beta_{k}\geq 0}Z_{DS} \end{array}$$ (8)

**Dual Subproblem:**

$$\begin{array}{c} \min_{t_{jk},v_{jk},y_{jk},\delta_{nk}}Z_{DS}=\{\sum_{k\in K}\sum_{j\in \Delta_{n}}\left[F_{j}v_{jk}+A_{j}y_{jk}+E_{j}\left(t_{jk}\right)\right]\\ +\sum_{n\in N}\sum_{k\in K}\lambda_{nk}[D_{nk}-\sum_{j\in \Lambda_{n}}t_{jk}-\sum_{m\in \Omega_{n}}B_{nm}\left(\delta_{mk}-\delta_{nk}\right)\\ +\sum_{m\in \Omega_{n}}K_{nm}\left(1-\cos\left(\delta_{mk}-\delta_{nk}\right)\right)]+\sum_{k\in K}\mu_{k}[D_{nk}+R_{k}-\sum_{j\in J}\bar{T_{j}}v_{jk}]\\ +\sum_{n\in N}\sum_{k\in K}\gamma_{nk}[c_{nm}-\sum_{m\in \Omega_{n}}B_{nm}\left(\delta_{mk}-\delta_{nk}\right)]\\ +\sum_{n\in N}\sum_{k\in K}\beta_{nk}[-\sum_{m\in \Omega_{n}}B_{nm}\left(\delta_{mk}-\delta_{nk}\right)-C_{nm}]\}. \end{array}$$ (9)

where *λ*_*nk*_ is the Lagrangian multiplier associated to a power balance constraint of node *n* in period *k*; *μ*_*k*_ is the Lagrangian multiplier associated to a spinning reserve requirement in period *k*; *γ*_*nk*_, *β*_*nk*_ are the Lagrangian multipliers associated to a transmission capacity limits of node *n* in period *k*. The above model is subject to Constraints (4), (6) and (7), called box constraints. Dualizing these constraints produces a dual subproblem that is less expensive to solve and speed up the solution of this subproblem. The algorithm initialized with a set of Lagrange multipliers, in this case, we defined these multipliers heuristically as a result of the knowledge of the original problem. Then, we use subgradient method to improve Lagrangian multipliers.

As the Lagrangian function constituted by the dualization of the complicating constraints is concave and non-differentiable, the AGS algorithm allows optimization of the dual function, since it is able to optimize such functions. The dual function can be decomposed into subproblems, one for each generation unit, however this procedure is not explored in this paper.

A brief summary of the subgradient method is given in Algorithm 1 below.

**Algorithm 1:** Subgradient method

**Input:** Instance of *Z*_*DS*_

**Output:** *S*^*sub*^: Infeasible solution.

1 **begin**

2  Set *k* ← 0, *h* ← 0 and *S*^*sub*^ ← ∅

3  Choose $\alpha^{0}\in R$ and *λ*^0^ ∈ [0, 2]

4  **repeat**

5   Compute *L*_*Z*_*DS*__(*α*^*k*^) and a vector $\vec{x}$ where it is achieved

6   Determine the subgradient direction *d*^*k*^ of the function *L*_*Z*_*DS*__ at *α*^*k*^

7   Determine step size *t*^*k*^ ← *λ*^*k*^(*UB* − *L*_*Z*_*DS*__(*α*^*k*^))/||*d*^*k*^||^2^

8   Update multiplier vector by using *α*^*k*+1^ ← max{0, *α*^*k*^ + *t*^*k*^ ⋅ *d*^*k*^}

9   **if** ($\vec{x}$ *better than* *S*^*sub*^) **then**

10    $S^{sub}\leftarrow \vec{x}$

11   **else**

12    *h* ← *h* + 1

13    **if** (*h is equal to some fixed number of iterations*) **then**

14     *λ*^*k*+1^ ← *λ*^*k*^/2

15   *k* ← *k* + 1

16  **until** (*termination criteria are not met*);

17  **return** *S*^*sub*^

### The adaptive gibbs sampling algorithm

The AGS algorithm is based on the Markov Chain Monte Carlo (MCMC) method combined with the one-dimensional Metropolis-Hastings algorithm and the multi-dimensional Gibbs sampler. This MCMC algorithm is called Metropolis-within-Gibbs (MWG) algorithm, and was suggested in [27]. The proposed optimization heuristic is a population-based method that generates a set of new potential solutions through a random process given by the MWG algorithm. The global information about the landscape is extracted through the population of solutions generated at each iteration. With the global information generated in the step described above, the random process identifies the most promising regions of the search space and then uses this information to generate another set of new potential solutions.

The main key of the proposed method is that the noise level of the random search can be adaptively controlled according to the landscape. Thus, the noise intensity allows exploring the entire search space, and the noise reduction allows the exploitation in the regions where local optima exist. In order to improve the obtained solutions, we consider coupling a local search into a random search process. The algorithm is well described and implement for unconstrained global optimization problems in [22]. A brief summary of the AGS method is given in Algorithm 2 below.

**Algorithm 2:** AGS(*M*, *c*^*o*^, *β*, *ε*, *λ*)

**Input:** *M*, *c*^*o*^, *β*, *ε*, *λ* ≔ AGS parameters.

**Output:** *S*^*best*^: An optimized and feasible solution.

1 **begin**

2  *S*^*best*^ ← ∅

3  $\vec{x}\leftarrow$ Initialization( )

4  **repeat**

5   $(\vec{X},\vec{\theta })\leftarrow$ Sampling($\vec{x}$)

6   $\vec{x}\leftarrow$ Selection($\vec{X}$)

7   $\vec{c}\leftarrow$ Update(*c*^*o*^, *λ*)

9   $\vec{x}\leftarrow$ Mutation($\vec{\theta },\varepsilon$)

10  **if** ($\langle \vec{\theta }\rangle >\beta$) **then**

11   $\vec{x}\leftarrow$ Intensification($\vec{x}$)

12  **if** ($\vec{x}$ *better than* *S*^*best*^) **then**

13    $S^{best}\leftarrow \vec{x}$

14  **until** (*termination criteria are not met*);

15  **return** *S*^*best*^

#### General form of AGS algorithm

Step 0 *Initialization:* Randomly select an initial solution $\vec{x}=\left(x_{1},x_{2},...,x_{n},...,x_{N}\right)$ within the feasible region and go to Step 1. Note that in this step we could also provide an initial solution obtained by other method. In this paper we use the solution that provides the subgradient method.
Step 1 *Sampling:* Generate candidate points for each variable by
  $$\begin{array}{c} x_{n}^{*}=x_{n}^{t}+c_{n}Z, \end{array}$$
  where *Z* is a standard normal random variable and *c*_*n*_ is a scale parameter. The candidate point will be accepted as the next value $x_{n}^{t+1}=x_{n}^{*}$ with probability
  $$\begin{array}{c} P=\min\{1,\frac{p(x_{1},x_{2},...,x_{n}^{*},...,x_{N})}{p(x_{1},x_{2},...,x_{n}^{t},...,x_{N})}\}. \end{array}$$
  If the candidate point is not accepted, then the current value of *x* is retained: $x_{n}^{t+1}=x_{n}^{t}$. Simulating one value in turn for each individual variable is called one cycle of Gibbs sampling where a new $\vec{x}$ vector solution is built. We can draw a population of *M* solutions by performing *M* Gibbs cycles. The output of the sampling step is a population $\vec{X}$ and a vector of acceptance rates $\vec{\theta }$. Finally, go to Step 2.
Step 2 *Selection:* Estimate a mode solution $\vec{x}$ for each variable in the population $\vec{X}$ and go to Step 3.
Step 3 *Update:* Adjust the scale parameters $\vec{c}$ by the following rule
  $$\begin{array}{c} c_{n}=c_{n}^{o}\tau^{-\lambda },\lambda >0, \end{array}$$
  where $c_{n}^{o}$ is a constant chosen so that initially the acceptance rates are close to zero. The actual iteration number, *τ*, is initialized at the beginning with *τ* = 1 and will be increased iteratively by *τ* = *τ* + 1. Go to Step 4.
Step 4 *Mutation:* Replace the variable value by a random value within the search space according to the following rule:
  $$\begin{array}{c} \text{If}\;\theta_{n}>\varepsilon ,\;\text{then}\;x_{n}=rand,\;c_{n}=c_{n}^{o}\;\text{and}\;\tau =1, \end{array}$$
  where *rand* is an uniform random variable within the feasible region. If $\langle \vec{\theta }\rangle >\beta$, where $\langle \vec{\theta }\rangle$ is an average of acceptance rates vector over all variables, go to Step 5; Otherwise return to Step 1.
Step 5 *Intensification:* Improve the solution $\vec{x}$ via a local search strategy. We can use an arbitrary local search method. In this paper, we use the Nelder-Mead method as a local search strategy [28]. Finally, return to Step 1.
Parameter settings The AGS parameters used in this paper are chosen such as to be a robust setting and therefore, in our experience, applicable to a wide range of optimization problems. The parameters used are: population size *M* = 100, initial scale parameters *c*^*o*^ = (0.1, …, 0.1), *β* = 0.7, *ε* = 0.95 and *λ* = 2.

The algorithm stops when the number of function evaluations is reached, or when the neighbor solution was not improved after the time period elapses. In Step 2, the global information generated by the population allows identifying the most promising (or more likely) regions of the search space. Therefore, the starting point for the next iteration will belong to such promising region. Note that in the Steps 3 and 4, the noise level of the random search can be adaptively controlled through acceptance rates. The acceptance rates provide information about the landscape. Values close to 1 on the acceptance rates allow to identify local optima and exploiting on them via local search. In addition, the mutation mechanism allows the escape and avoids being trapped in local optima, exploring other regions of the search space. In this way, the noise intensity allows exploring the entire search space, and the noise reduction allows exploitation in the promising regions where local optima exist.

#### AGS algorithm for STGS problem

Before using the AGS algorithm to solve the STGS problem, we must define the representation of a solution. A solution $\vec{x}$ is composed of two parts. The first part contains continuous variables, and the second contains integer variables, that is, $\vec{x}=\vec{x_{c}}\cup \vec{x_{b}}$. In the STGS problem, $\vec{x_{c}}=\left\{t_{jk},\delta_{nk}\right\}$ contains the variables of power output of plant *j* in period *k* and the angle of node *n* in period *k*, respectively. $\vec{x_{b}}=\left\{v_{jk},y_{nk}\right\}$ contains the variables *v*_*jk*_ that represents if plant *j* is committed in period *k* and *y*_*nk*_ represents if plant *j* is started up at the beginning of period *k*.

In the initialization process, Step 3 in Algorithm 2, an initial solution is provided by the method RL (see Section “Lagrangian Relaxation Framework”). Next, in Step 4, the integer variables are fixed, and continuous variables are modified by the Gibbs cycles. The integer variables can be modified after Step 9 in Algorithm 2, by the following rule:

$$\begin{array}{c} y_{nk}=\{\begin{array}{ccc} 0\; & \text{if} & y_{nk-1}=1,\\ 0\;\text{or}\;1\; & \text{if} & y_{nk-1}=0. \end{array} \end{array}$$

After modifying the *y*_*nk*_ value, *v*_*jk*_ will make a copy of the value, that is, *v*_*nk*_ = *y*_*nk*_.

The Algorithm 3 shows the exchange of information between the Lagrangian relaxation scheme and AGS algorithm.

**Algorithm 3:** *AGS*_*m*_(*M*, *c*^*o*^, *β*, *ε*, *λ*)

**Input:** *M*, *c*^*o*^, *β*, *ε*, *λ* ≔ AGS parameters.

**Output:** *S*^*best*^: An optimized and feasible solution.

1 **begin**

2  *S*^*best*^ ← *Subgradient*()

3  $\vec{x}\leftarrow S^{best}$

4  **repeat**

5   $(\vec{X},\vec{\theta })\leftarrow$ Sampling($\vec{x}$)

6   $\vec{x}\leftarrow$ Selection($\vec{X}$)

7   $\vec{c}\leftarrow$ Update(*c*^*o*^, *λ*)

8   $\vec{x}\leftarrow$ Mutation($\vec{\theta },\varepsilon$)

9   Generate *k* at random, *k* = 1, 2, …*K*

10   **if** (*y*_*nk*−1_ = 1) **then**

11    *y*_*nk*_ ← 0

12    *v*_*nk*_ ← *y*_*nk*_

13   **if** (*y*_*nk*−1_ = 0) **then**

14    *r* ← *rand*(0, 1)

15    *y*_*nk*_ ← *r*

16    *v*_*nk*_ ← *y*_*nk*_

17   **if** ($\langle \vec{\theta }\rangle >\beta$) **then**

18    $\vec{x}\leftarrow$ Intensification($\vec{x}$)

19   **if** ($\vec{x}$ *better than* *S*^*best*^) **then**

20    $S^{best}\leftarrow \vec{x}$

21  **until** (*termination criteria are not met*);

22  **return** *S*^*best*^

## Computational experience

The AGS algorithm was developed in C++ and simulated on a desktop computer with an AMD Phenom TM II N970 Quad-Core with a 2.2 GHz processor and 8 GB RAM. For Lagrangian Relaxation, the mathematical model of STGS was implemented in the modeling environment GAMS using the solver DICOPT (see [26] and [29]) for solving the MINLP problems (dual subproblems and full STGS scale model).

Three test systems are presented to evaluate the performance of the proposed AGS algorithm in the engineering optimization problems. The test cases used in the experimentation are power systems of 24, 104 and 118 units with a planning horizon of 24 h.

**Fig 1:**
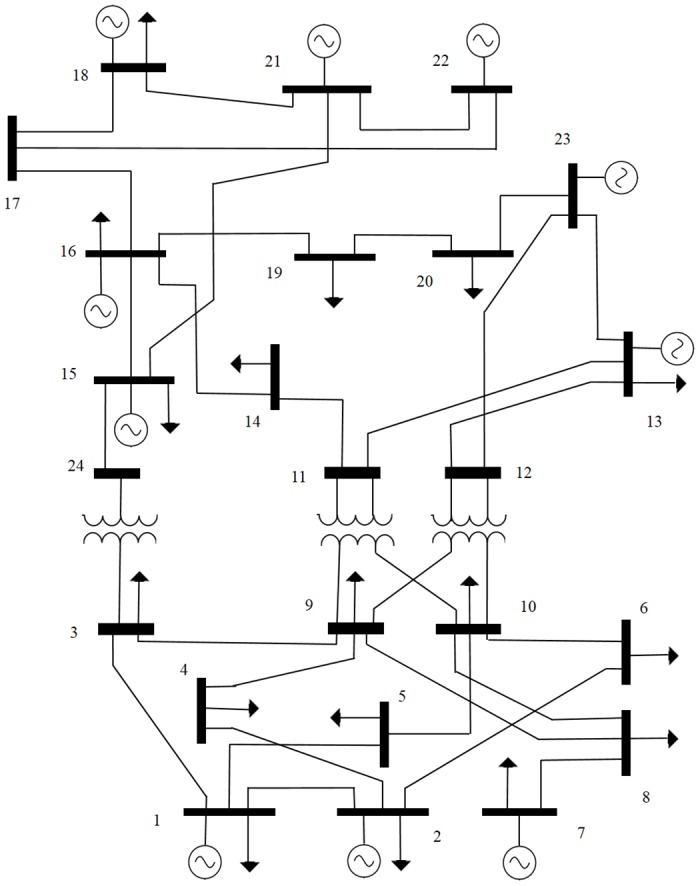
IEEE 24-bus. Layout of IEEE 24-bus test system.

**Fig 2:**
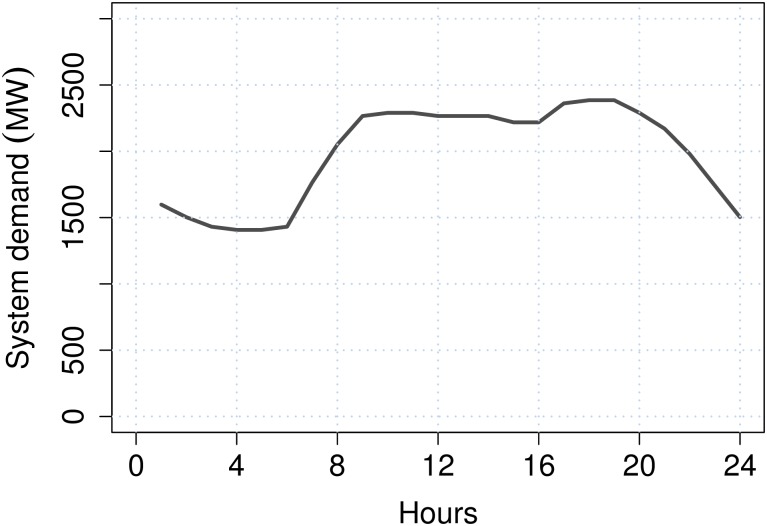
System demand profile.

In order to illustrate the structure of test systems, we choose the basic information of the IEEE 24-bus test system. The single-line diagram of the IEEE 24-bus test system with 24 nodes, 24 thermal units and 38 transmission lines is shown in Fig 1. The data used in the IEEE-24 bus system were based on the original system which is available at [30]. The data of the minimum and maximum capacities generating units are presented in Table 1. Table 2 lists the costs, initial state and power output of each generating unit at time 0. In Table 3 and Fig 2 the load profile is illustrated. The node location of the loads, as well as load at each node as a percentage of the total system demand are presented in Table 4. The transmission lines data is given in Table 5. The lines are characterized by the nodes that are connected, as well as the reactance and the capacity of each line.

**Table 1:** Technical data of generating units.

Unit	Node	*P*_max_	*P*_min_	*R*^+^	*R*^−^	*RU*	*RD*	*UT*	*DT*
		MW	MW	MW	MW	MW/min	MW/min	h	h
Unit 18	18	400	100	0	0	6.67	6.67	1	1
Unit 21	21	400	100	0	0	6.67	6.67	1	1
Unit 1	1	152	30.4	40	40	2	2	8	4
Unit 2	2	152	30.4	40	40	2	2	8	4
Unit15b	15	155	54.25	30	30	3	3	8	8
Unit 16	16	155	54.25	30	30	3	3	8	8
Unit 23a	23	310	108.5	60	60	3	3	8	8
Unit 23b	23	350	140	40	40	4	4	8	8
Unit 7	7	350	75	70	70	7	7	8	8
Unit 13	13	591	206.85	180	180	3	3	12	10
Unit 15a	15	60	12	60	60	1	1	4	2
Unit 22	22	300	300	0	0	5	5	0	0

**Table 2:** Costs and initial state of generating units.

Unit	*C*	*C*_*u*_	*C*_*d*_	*C*_*su*_	*P*_*ini*_	*U*_*ini*_	*T*_*ini*_
	$/MWh	$/MWh	$/MWh	$	MW	0/1	h
Unit 18	5.47	0	0	0	320	1	50
Unit 21	5.47	0	0	0	320	1	16
Unit 1	13.32	15	14	1430.4	121.6	1	22
Unit 2	13.32	15	14	1430.4	121.6	1	22
Unit15b	10.52	16	14	312	0	0	-2
Unit 16	10.52	16	14	312	124	1	10
Unit 23a	10.52	17	16	624	248	1	10
Unit 23b	10.89	16	14	2298	280	1	50
Unit 7	20.7	10	9	1725	0	0	-2
Unit 13	20.93	8	7	3056.7	0	0	-1
Unit 15a	26.11	7	5	437	0	0	-1
Unit 22	0.00	0	0	0	240	1	24

**Table 3:** Load profile.

Hour	System Demand	Hour	System Demand
	MW		MW
1	1598.252	13	2266.178
2	1502.834	14	2266.178
3	1431.27	15	2218.469
4	1407.416	16	2218.469
5	1407.416	17	2361.596
6	1431.27	18	2385.45
7	1765.233	19	2385.45
8	2051.487	20	2290.032
9	2266.178	21	2170.76
10	2290.032	22	1979.924
11	2290.032	23	1741.379
12	2266.178	24	1502.834

**Table 4:** Node location and distribution of the total system demand.

Load	Node	% of system load	Load	Node	% of system load
Load 1	1	3.8	Load 10	10	6.8
Load 2	2	3.4	Load 13	13	9.3
Load 3	3	6.3	Load 14	14	6.8
Load 4	4	2.6	Load 15	15	11.1
Load 5	5	2.5	Load 16	16	3.5
Load 6	6	4.8	Load 18	18	11.7
Load 7	7	4.4	Load 19	19	6.4
Load 8	8	6	Load 20	20	4.5
Load 9	9	6.1			

**Table 5:** Reactance and capacity of transmission lines.

From	To	Reactance	Capacity	From	To	Reactance	Capacity
		p.u.	MWA			p.u.	MWA
1	2	0.0146	175	11	13	0.0488	500
1	3	0.2253	175	11	14	0.0426	500
1	5	0.0907	350	12	13	0.0488	500
2	4	0.1356	175	12	23	0.0985	500
2	6	0.205	175	13	23	0.0884	500
3	9	0.1271	175	14	16	0.0594	500
3	24	0.084	400	15	16	0.0172	500
4	9	0.111	175	15	21	0.0249	1000
5	10	0.094	350	15	24	0.0529	500
6	10	0.0642	175	16	17	0.0263	500
7	8	0.0652	350	16	19	0.0234	500
8	9	0.1762	175	17	18	0.0143	500
8	10	0.1762	175	17	22	0.1069	500
9	11	0.084	400	18	21	0.0132	1000
9	12	0.084	400	19	20	0.0203	1000
10	11	0.084	400	20	23	0.0112	1000
10	12	0.084	400	21	22	0.0692	500

The 104-bus system data were extracted from [24] and correspond to the energy system of mainland Spain. The data used in the IEEE 118-bus system were extracted from the original system which is available at [31]. This data contains information about reactance and capacitance of the transmission lines, the demand profile, and costs of the generation.

### Results

The computational complexity of the problem is shown in Table 6. This complexity depends on the number of thermal units connected in each test system. This table shows the number of variables and constraints for each test system.

**Table 6:** Model complexity.

Test Systems	Constraints	Variables	
		Integer	Continuous
IEEE 24-bus	4800	1584	1368
104-bus	15312	3408	4200
IEEE 118-bus	15672	2592	4128

**Fig 3:**
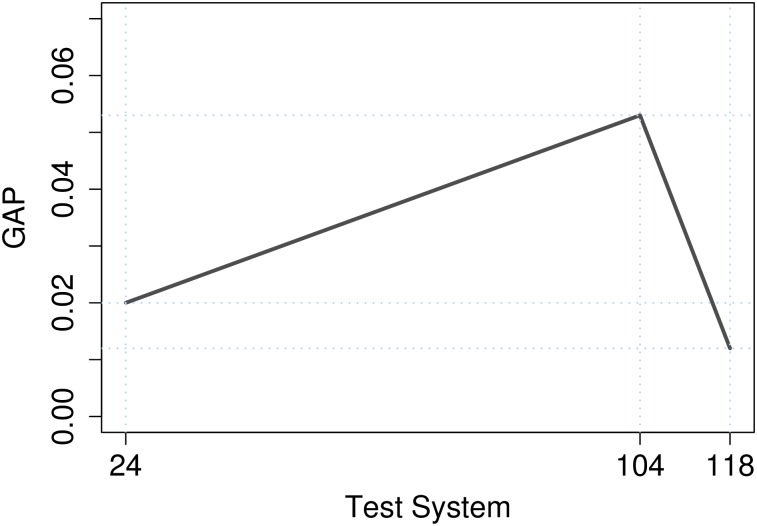
Average gap for the three test systems using AGS.

As the AGS algorithm is a stochastic approach, 20 runs are executed for AGS on each test case, and the average costs of the 20 runs are determined. The comparison results of AGS algorithm and full-scale outer approximation commercial solver (DICOPT) are shown in Table 7. These results show that AGS solutions are very close to the GAMS solutions z*. The GAP in all cases is less than 0.05%. Fig 3 shows the average Gap for the three test systems using AGS. Standard deviation is also shown in Table 7. The GAP between the best solution through GAMS solver and the AGS is calculated by *Z*_*AGS*_ GAP = (z* − *z*_*AGS*_)/z*.

**Table 7:** Comparison between AGS and DICOPT.

Test Systems	AGS algorithm			DICOPT	
	*Z*_AGS_ GAP (%)	Std. Dev.	Time (sec)	z* (in $)	Time (sec)
IEEE 24-bus	0.020	8.07e-03	60	5681	360
104-bus	0.053	2.10e-05	230	67457	1740
IEEE 118-bus	0.012	3.18e-03	180	1215	1260

**Fig 4:**
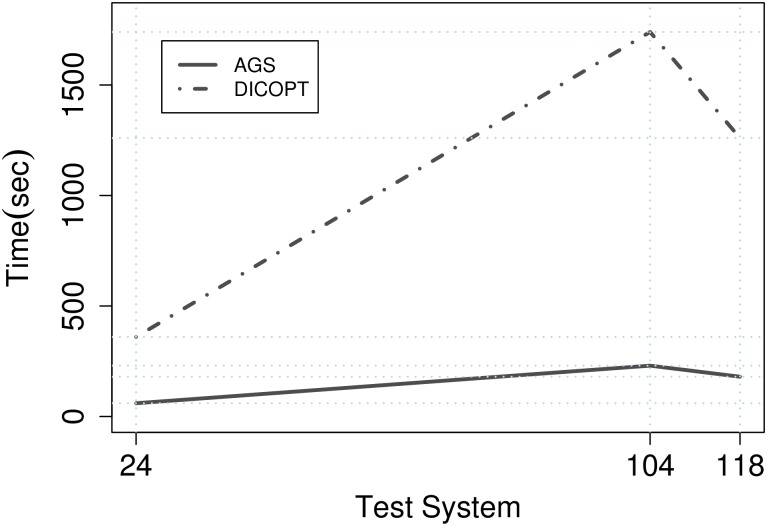
Growth of computation time with respect to system size.

Additionally, it can be seen in Table 7, that the CPU time of proposed algorithm is much less than DICOPT. Fig 4 shows the time evolution of the comercial solver DICOPT versus AGS algorithm. The maximum CPU time of AGS algorithm in comparison with DICOPT is 230 seconds. The minimum time improvement of AGS algorithm is obtained in the IEEE 24-bus test system. The total CPU time required to carry out these tests systems was about 180 seconds.

## Conclusions and future work

In this paper, we present a novel optimization method which we call Adaptive Gibbs Sampling (AGS) algorithm to address a Short Term Generation Scheduling with network constraints, which determines the startup and shutdown schedules of thermal units over a given planning horizon. The proposed heuristic is a population-based method that generates a set of new potential solutions via random search strategy. The random search is based on a Markov Chain Monte Carlo method. The key to the proposed method is that the noise level of the random search is adaptively controlled in order to exploring and exploiting the entire search space. In order to improve the solutions, we consider coupling a local search into a random search process. This paper proposes an enhanced method that combines Lagrangian relaxation and AGS to solve STGS. The AGS algorithm is based on infeasible solutions calculated for LR algorithm.

We evaluated the performance of AGS algorithm against a full-scale outer approximation commercial solver in order to compare the quality of the solutions provided by the proposed method. Several groups of instances are tested to evaluate the performance of the proposed heuristic. The experimental results show that AGS algorithm is robust, as it is capable of finding reasonable quality solutions for this engineering optimization problem. Our AGS method converges to the near-optimal solution at a faster rate than the direct solution obtained by the solver DICOPT. In addition, AGS produces much tighter bounds on the optimal solution values than standard Lagrangian Relaxation.

In the future, we will extend the experimentation in other test systems with different structures in order to evaluate the performance of the proposed algorithm, and compare their results against other heuristic methods.

## Supporting information

S1 File Datafile for the IEEE 24-bus test system. (TXT)

S2 File Datafile for the IEEE 118-bus test system. (TXT)
